# Radish Red Anthocyanins Extract Attenuated High-Fat Diet-Induced Aging in Association with Improving Antioxidant Defense and Gut Microbiota–Metabolite Changes in Drosophila Melanogaster

**DOI:** 10.3390/nu18213499

**Published:** 2026-10-24

**Authors:** Zhenxia Li, Can Xu, Qingjing Liao, Fayin Ye, Fasheng Lv, Cai Peng, Zhenyu Chen, Lin Lei

**Affiliations:** 1 College of Food Science, Southwest University, No. 2 Tiansheng Road, Chongqing 400715, China; 2 Southeast Chongqing Academy of Agricultural Sciences, Fuling Academy of Southwest University, Chongqing 408000, China; 3 School of Food Science, Guangdong Pharmaceutical University, Guangzhou 510006, China

**Keywords:** radish red anthocyanins, *Drosophila melanogaster*, high-fat diet, oxidative stress, gut microbiota, metabolomics

## Abstract

Background: Radish red anthocyanins (RRA) extract, derived from geographical-indication Yanzhi radish from Fuling, Chongqing, China, was investigated to attenuate high-fat diet- induced aging in *Drosophila melanogaster*. RRA extract, rich in acylated pelargonidin- and cyanidin-based anthocyanins, extended lifespan without reducing food intake, improved reproductive and locomotor performance, raised superoxide dismutase and catalase activities, whereas reduced malondialdehyde accumulation. Mthoths: Using a *Drosophila melanogaster* model, we investigated the anti-aging effects of RRA extract and its potential underlying mechanisms. Results: It enhanced resistance to starvation, cold, and heat stress, reduced age-associated intestinal leakage, and regulated antioxidant- and autophagy-related genes, particularly manganese superoxide dismutase (SOD2) and autophagy-related 8a (Atg8a). Gut microbiota analysis showed high-dose RRA supplementation selectively increased *Pseudomonas*-related amplicon sequence variants 30 and 31. Untargeted metabolomics showed variations in glycerophospholipid, sphingolipid, nucleotide, and amino acid metabolism. Correlation results linked these *Pseudomonas*-related bacteria with lipid/nucleotide-related metabolites and stress resistance. Conclusions: RRA extract alleviated diet-induced aging through antioxidant defense, intestinal protection, and gut microbiota–metabolite modulation, providing a research basis for high-value utilization of Yanzhi radish anthocyanins.

## 1. Introduction

Promoting healthy aging requires nutritional strategies that help preserve physiological function and reduce the burden of age-related metabolic disorders. Excessive dietary fat intake can increase lipid load and disrupt metabolic homeostasis, contributing to oxidative stress, chronic inflammation, and impaired intestinal function [1,2,3,4]. These disturbances are associated with reduced survival and aging-related physiological decline [2,5]. Dietary interventions targeting these changes may help preserve functional capacity under nutritional stress. Identifying food-derived bioactive compounds that alleviate the adverse effects of excessive fat intake may therefore provide an experimental basis for nutritional approaches to support healthy aging.

Oxidative stress, impaired cellular maintenance, and intestinal dysfunction are interrelated processes which contribute to age-related functional decline. Excessive reactive oxygen species (ROS) can overwhelm cellular antioxidant capacity, impairing cell function through oxidative damages of lipids, proteins, and nucleic acids. Cells maintain redox balance through endogenous enzymatic systems, in which superoxide dismutase (SOD) converts superoxide radicals into hydrogen peroxide and catalase (CAT) facilitates its decomposition. Autophagy complements these processes by clearing damaged organelles and macromolecules to maintain cellular quality [6]. Maintenance of physiological homeostasis also depends on a functional intestinal barrier and metabolic interactions between the host and its gut microbiota. Therefore, evaluating antioxidant defenses, intestinal function, and microbial and metabolic responses may help elucidate how dietary bioactive compounds influence functional outcomes under nutritional stress.

Anthocyanins are dietary flavonoids widely found in plants and have attracted interest for their potential to support health through antioxidant, anti-inflammatory, and metabolic effects, including interactions with the gut microbiota [7,8,9]. Studies using cranberry [10], purple sweet potato [2], black rice [11], and blackcurrant [12] extracts rich in anthocyanins provide evidence for their potential role in healthy aging. These studies have reported lifespan extension or improvements in aging-related phenotypes in *Drosophila melanogaster*, accompanied by changes in oxidative stress, stress resistance, intestinal homeostasis, or aging-related signaling. However, the biological effects of anthocyanins vary substantially depending on their structural characteristics and food sources.

Yanzhi radish (*Raphanus sativus* L.), cultivated in Fuling, Chongqing, China, is a source of anthocyanins used to manufacture the natural colorant radish red, a China-approved natural food colorant (CNS 08.117). Radish red anthocyanins (RRA) contain acylated pelargonidin- and cyanidin-based derivatives, with acylation contributing to pigment stability under relevant processing conditions [13,14]. These characteristics support interest in their further value-added utilization as food ingredients, although pigment stability does not establish superior biological efficacy relative to anthocyanins from other sources. Previous research demonstrated enzymatic hydrolysis increased the antioxidant capacity of RRA in vitro and their antiproliferative activity against human hepatocellular carcinoma MHCC97L cells [15]. RRA could reduce oxidative damage and apoptotic cell death following palmitic acid exposure in rat aortic endothelial cells, with involvement of the p38 MAPK pathway [16]. These findings support further investigation of RRA under dietary stress. However, to our knowledge, whether RRA affects lifespan and preserves physiological performance during aging in high-fat fed *Drosophila melanogaster* have not previously been reported. Therefore, investigating these effects may provide an experimental basis for exploring potential functional applications of RRA beyond coloration.

*Drosophila melanogaster* provides an ideal model for investigating how dietary interventions influence longevity and physiological performance during aging. High-fat feeding can shorten lifespan and impair locomotor activity, stress resistance, and intestinal barrier function, making this model useful for evaluating dietary components against diet-induced functional decline [2,5]. In this study, flies received a lard-enriched basal diet to induce physiological changes associated with aging. The chemical profile of RRA extract and its effects on lifespan, functional phenotypes, stress responses, and intestinal barrier integrity were investigated. Antioxidant- and autophagy-related gene expression, gut microbiota composition, and intestinal metabolite profiles were also examined to explore biological responses associated with these effects. We aimed to to assess RRA extract as a dietary candidate for promoting healthy aging and guide further nutritional studies in mammals and humans.

## 2. Materials and Methods

### 2.1. Materials and Chemicals

Danqing Biotechnology Co., Ltd. (Chongqing, China) supplied RRA extract. Anthocyanins standards were obtained from SHANGHAI ZZBIO CO., Ltd. (Shanghai, China). Shanghai Yuanye Biotechnology Co., Ltd. (Shanghai, China) provided MDA (R21869) assay kit. Assay kits of SOD (BC0175) and CAT (BC0200) activity were provided by Beijing Solarbio Science & Technology Co., Ltd. (Beijing, China). Formic acid of LC/MS grade was supplied by Thermo Fisher Scientific (Waltham, MA, USA). Methanol and acetonitrile of LC/MS grade was sourced from Merck Millipore (Darmstadt, Germany).

### 2.2. UPLC-MS/MS Analysis of RRA Extract

Extraction and determination were performed according to a reported method with slight modifications [17]. 20 mg of RRA extract was dissolved in 1 mL of aqueous methanol (70%, *v*/*v*) supplemented with formic acid (0.1%, *v*/*v*). After homogenization for 15 min at 45 Hz, the sample was sonicated at 4 °C for 30 min, followed by centrifugation at 12,000 rpm for 15 min. The collected supernatant was passed through a 0.22 μm membrane before UPLC–MS/MS analysis.

An ACQUITY I-Class UPLC instrument (Waters, Milford, MA, USA) equipped with an ACQUITY UPLC HSS T3 column (100 × 2.1 mm, 1.8 μm, Waters, USA) was used to separate the compounds. Samples were injected at a volume of 2 μL, with the column held at 35 °C. Elution was carried out at 0.4 mL/min using water and acetonitrile as solvents A and B, respectively, each containing 0.1% (*v*/*v*) formic acid. Supplementary Table S1 shows the gradient elution procedure.

The UPLC eluent was introduced into a QTRAP 6500+ triple quadrupole mass spectrometer (SCIEX, Marlborough, MA, USA) fitted with an IonDrive Turbo V electrospray (SCIEX, Marlborough, MA, USA) ionization source. Mass spectrometer data were acquired using multiple reaction monitoring mode. Each standard was prepared at 20 ug/mL using a methanol-water mixture (1:1, *v*/*v*). MS parameters were optimized using anthocyanins standards. The source temperature was maintained at 550 °C, with spray voltages of +5500 V and −4500 V for positive and negative ionization, respectively. Gas pressures were 35 psi for the curtain gas, 50 psi for ion source gas 1, and 55 psi for ion source gas 2. SCIEX Analyst Workstation (version 1.7.2) and SCIEX OS (version 2.0.1) were used for data collection and quantitative analysis.

### 2.3. Fly and Diets

**Figure 1:**
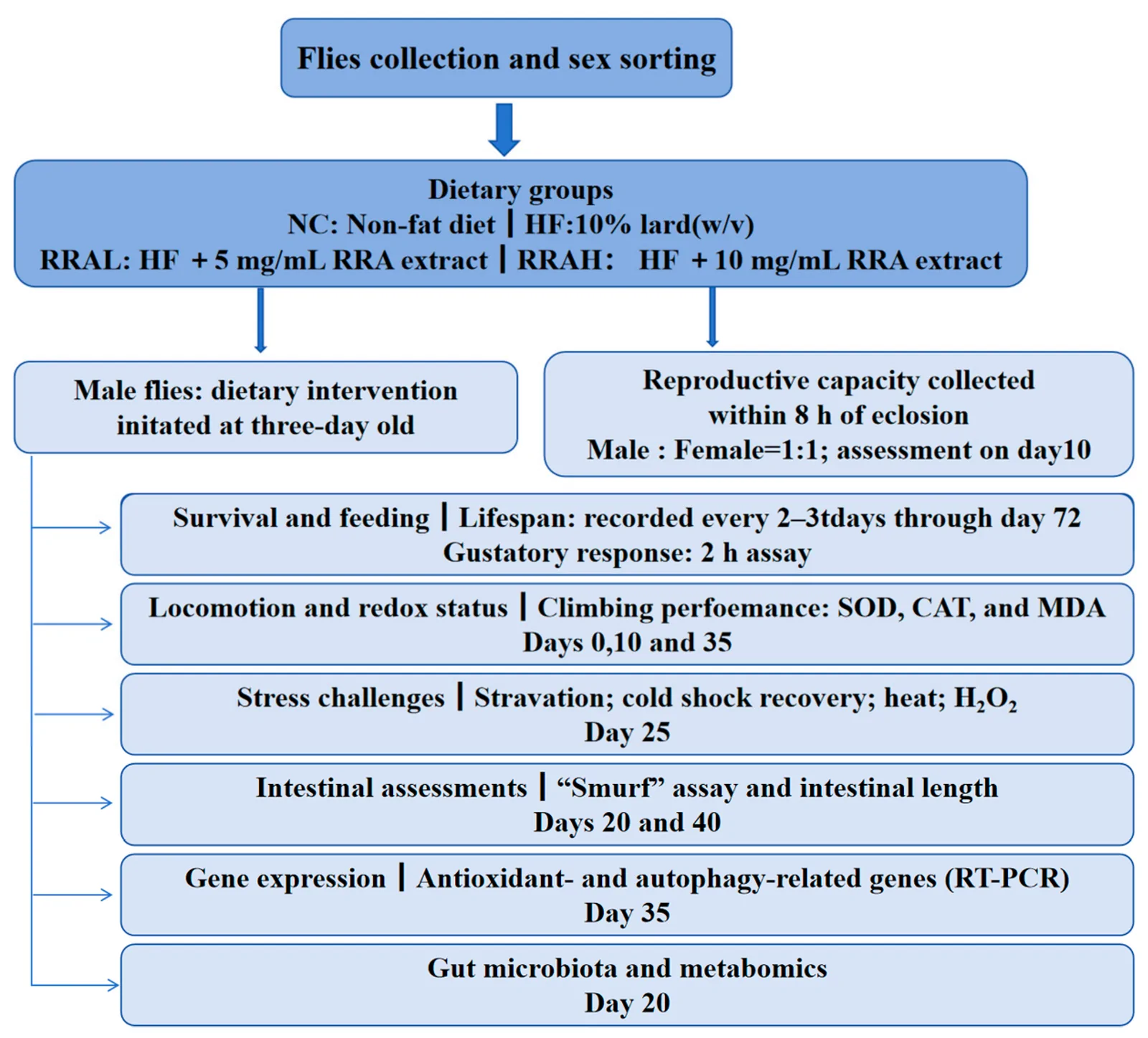
Overview of the experimental design for evaluating the effects of radish red anthocyanin (RRA) extract in high-fat-fed *Drosophila melanogaster*.

Wild-type *Drosophila melanogaster* of *W*^1118^ strain was supplied by Fungene Biotechnology Co., Ltd. (Nantong, China). The standard non-lard diet (NC) was formulated as previously reported with modifications [2]. The basal formulations was prepared by mixing corn flour (26 g), glucose (26 g), yeast (5 g), and agar (3 g) with 325 mL of distilled water. 20 mL of 1% (*w*/*v*) ethyl 4-hydroxybenzoate solution prepared in 70% (*v*/*v*) ethanol was added to inhibit mold growth. The NC diet contained Tween-20 at 1% (*v*/*v*). The high-fat diet (HF) was prepared by adding lard to the NC formulation at a final concentration of 10% (*w*/*v*) and mixing thoroughly. Two experimental diets were similarly prepared by supplementing HF diet with 5 mg/mL RRA extract (RRAL) or 10 mg/mL RRA extract (RRAH). For stock maintenance, 10 mL of NC diet was dispensed into each vial, while 5 mL of the corresponding experimental diet was added to each vial for experimental flies. The RRA extract concentrations of 5 and 10 mg/mL diet were selected with reference to previous anthocyanin intervention studies in *Drosophila melanogaster* [2,10] to explore effects on lifespan and aging-related phenotypes under high-fat feeding. Differences in extract composition, however, preclude assuming equivalent anthocyanin exposure or biological potency across studies. Based on the calculated energy content of the experimental diets, these concentrations corresponded to 3.47 and 6.94 g RRA extract/1000 kcal, respectively. The adult specific proposed level (SPL) for proanthocyanidins in the Dietary Reference Intakes for China (2023) is 200 mg/day, corresponding to 100 mg/1000 kcal at an assumed energy intake of 2000 kcal/day. Although the RRA extract supplementation levels were numerically higher, this comparison therefore provides dietary context rather than a basis for dose selection, human-equivalent exposure, or safety assessment. Flies were maintained at 25 ± 1 °C and a relative humidity of 60–70%, with alternating 12 h periods of light and darkness. Three-day old male flies were used, except in the reproductive capacity assay, to minimize potential sex-related variation. A summary of all assays and the experimental workflow are provided in Supplementary Table S2 and Figure 1.

### 2.4. Lifespan Assay

Flies were randomly assigned to four groups and maintained on corresponding diets, with 200 flies per group housed in 10 vials (20 flies each vial). Mortality was recorded at intervals of 2–3 days, while surviving flies were moved to new vials supplied with respective diets. The lifespan assay lasted for 72 days.

### 2.5. Gustatory Assay

A dye-based feeding assay was conducted to assess whether reduced food intake might cause the observed survival benefits [2]. A total of 240 flies were initially maintained on the NC diet for 6 days (20 flies per vial). Following 24 h of food deprivation on Kimwipes dampened with distilled water, flies were randomly assigned to four groups. Each gropup received its respective diet containing 0.2% sulforhodamine B sodium salt. Each group contained 60 flies housed in three vials (20 flies per vial). Abdominal staining served as an indicator of food intake and was rated from 0 (a colorless abdomen) to 5 (a fully red abdomen).

### 2.6. Reproductive Capacity Assay

Flies were collected within 8 h of eclosion. They were separated by sex and then randomly allocated to four dietary groups, each comprising 50 males and 50 females flies. During the rearing period, the flies were transferred every 2–3 days to fresh vials containing the same diet. After 5 days, 10 males and 10 females from each group were combined in a vial and maintained on their assigned diet for an additional 5 days. The parental flies were then removed, and the progeny were allowed to develop for 10 days. Pupae adhering to the vial walls were counted for each group.

### 2.7. Climbing Ability Test

Locomotor performance following RRA supplementation was assessed using the climbing assay. Each dietary group comprised 200 flies housed in 10 vials (20 flies per vial). Flies were given 10 s to climb. Those reached or exceeded the 7 cm mark were counted. Triplicate were conducted on days 0, 10, and 35.

### 2.8. Antioxidant Activity Test

Each group included 200 flies housed in 10 vials, 20 flies per vial. Before sample preparation, flies were moved to food-free vials for 2 h. After weighing, flies homogenized in 2 mL of ice-cold phosphate-buffered saline (PBS). Homogenates were centrifuged at 1200× *g* for 10 min at 4 °C, and the recovered supernatants were used to measure SOD and CAT activities and MDA content with commercial kits following the manufacturers’ protocols. Measurements were conducted in triplicate on days 0, 10, and 35.

### 2.9. Stress Challenge Tests

Day 25 was selected for stress challenge tests based on the survival results. Male flies from each dietary group, distributed among 10 vials with 20 individuals per vial, were tested using a modified method [18].

#### 2.9.1. Starvation Stress

Flies were placed in vials supplied with 1.5% agar as a moisture source without food. Mortality was assessed at 5 h intervals until no flies remained alive.

#### 2.9.2. Cold Shock Recovery

After a 30 min acclimation period in empty vials at 25 ± 1 °C, flies were exposed to 0 °C for a further 30 min in a cold bath. Then, flies were returned to empty vials maintained at 25 ± 1 °C and the recovery time was recorded.

#### 2.9.3. Heat Stress

After a 30 min acclimation period in empty vials at 25 ± 1 °C, flies was placed in a temperature-controlled incubator at 37 °C. Mortality was monitored at 10 min intervals until no individuals remained alive.

#### 2.9.4. H_2_O_2_ Challenge Test

H_2_O_2_ was used to induce oxidative stress. Flies underwent 2 h of food deprivation in vials lined with filter paper moistened with distilled water. Then flies were moved to empty vials containing filter paper soaked with 1 mL of 30% H_2_O_2_ in 6% glucose solution. Mortality was assessed at 4 h intervals until no flies remained alive.

### 2.10. “Smurf” Assay

On days 20 and 40, intestinal permeability was assessed using the Smurf assay [19]. Each group comprised 200 flies housed among 10 vials, with 20 individuals per vial. Following 2 h of food deprivation in empty vials, flies received a 12 h exposure to filter paper soaked in a 2.5% (*w*/*v*) blue dye solution. The proportion of individuals exhibiting blue coloration (“Smurfs”) was calculated for each group.

### 2.11. Intestinal Length Analysis

Each group comprising 200 flies housed among 10 vials, with 20 individuals per vial, were moved to empty vials for 2 h before anesthetized by carbon dioxide. Following dissection, the intestines underwent fixation for 40 min in 4% paraformaldehyde, rinsed with ice-cold PBS, and mounted in 70% glycerol for microscopic observation [20]. Images of the dissected intestines were analyzed in ImageJ (version 1.54j) to determine their lengths [21]. The assay was performed on days 20 and 40.

### 2.12. Real-Time PCR

The relative mRNA levels of copper–zinc superoxide dismutase (SOD1), manganese superoxide dismutase (SOD2), CAT, autophagy-related 1 (Atg1), autophagy-related 5 (Atg5), and autophagy-related 8a (Atg8a) were normalized to ribosomal protein 49 (*rp49*) (Supplementary Table S3). Relative gene expression was calculated using the 2^−ΔΔCt^ method. Briefly, on day 35, total RNA was extracted from 60 flies per group housed in 3 vials (20 flies per vial) using TRIzol reagent (TransGen Biotech Co., Ltd., Beijing, China) [10]. RNA concentration and purity were quantified spectrophotometrically. cDNA was synthesized using the HiScript III 1st Strand cDNA Synthesis Kit (+gDNA wiper) (Vazyme, Nanjing, China). Transcript quantification was conducted on a LineGene 9600 Plus fluorescence quantitative PCR detection system (Bioer, Hangzhou, China) with SupRealQ Ultra Hunter SYBR qPCR Master Mix (U+) (Vazyme, Nanjing, China).

### 2.13. Analysis of Gut Microbiota

On day 20, male flies were rinsed with 75% (*v*/*v*) ethanol and washed with sterile PBS before dissection. Midguts were dissected in sterile PBS using sterile forceps and collected in pre-cooled PBS. Genomic DNA was isolated with the E.Z.N.A.^®^ soil DNA Kit (Omega Bio-tek, Norcross, GA, USA). The V3-V4 region of Bacterial 16S rRNA gene were amplified with primers 338F (5′-ACTCCTACGGGAGGCAGCA-3′) and 806R (5′-GGACTACHVGGGTWTCTAAT-3′). Majorbio Bio-Pharm Technology Co., Ltd. (Shanghai, China) performed sequencing using an Illumina NextSeq 2000 instrument (San Diego, CA, USA). The resulting sequences were then processed by Naive bayes, clustering them into amplicon sequence variants (ASVs) based on a sequence similarity threshold of ≥97%. Taxonomic information was obtained in QIIME 2 (v.2024.10), referencing SILVA 16S rRNA database (138.2). Community richness (Sobs, Chao, and ACE) and diversity (Shannon and Simpson) were assessed using mothur (version 1.30.2). Following normalization of sequencing read counts across samples, bacterial relative abundances were summarized at the phylum and genus levels. Rarefaction curves based on Sobs and Shannon indices were examined to assess whether sequencing coverage adequately represented the sampled bacterial communities. Beta diversity was visualized by principal coordinate analysis (PCoA) of unweighted_unifrac distance using the Vegan package (version 2.5-3). Group differences in community structure were tested by permutational multivariate analysis of variance (PERMANOVA). Discriminatory taxa was determined using linear discriminant analysis effect size (LEfSe), with an LDA score greater than 2.0 as the selection threshold.

### 2.14. Analysis of Gut Metabolites

On day 20, male flies were rinsed with 75% (*v*/*v*) ethanol and washed with sterile PBS before dissection. Midguts were dissected in sterile PBS using sterile forceps, collected in pre-cooled PBS, and immediately flash-frozen in liquid nitrogen. Then, each homogenized sample was as described in a previous study [22]. The gradient elution program for LC-MS/MS analysis is provided in Supplementary Table S4. The flow rate was 0.40 mL/min, the column temperature was 40 °C, and the injection volume was 3 μL. Differential metabolites were visualized to metabolic pathways using the Kyoto Encyclopedia of Genes and Genomes (KEGG) database [23]. Metabolic pathway analysis of *Drosophila melanogaster* was further performed to identify pathways most relevant to changes in metabolite abundance.

### 2.15. Statistical Analysis

Statistical significance among groups was analyzed by SPSS software (version 26.0, Chicago, IL, USA) using one-way analysis of variance followed by Turkey’s multiple range test. Microbiome profiling included three biological replicates per dietary group, each comprising pooled samples from 60 *Drosophila melanogaster*. Metabolomic profiling included six biological replicates per dietary group, each comprising pooled samples from 145 *Drosophila melanogaster*. ASVs and metabolites relative abundance was visualized in heatmaps among all groups, with average-linkage hierarchical clustering. Relationships among selected ASVs, stress-resistance measures, and intestinal metabolites were examined using Spearman’s rank correlations. Data were presented as mean ± standard deviation. A *p* value < 0.05 was considered statistically significant.

## 3. Results and Discussion

### 3.1. Anthocyanins Profile of RRA Extract

The chemical composition of RRA extract was first characterized to establish the phytochemical basis for its biological effects. 48 compounds were determined in RRA extract, among which anthocyanins accounted for 99.76% of the total detected compounds. The major anthocyanins were 11 acylated, 7 purely glycosylated, and 1 aglycone form (Supplementary Table S5). Acylated anthocyanins generally show improved stability compared with non-acylated forms, which may favor their biological activity during dietary intervention. All identified peaks exhibited characteristic fragment ions at *m*/*z* 286.9 and 271.1 in positive ionization mode, indicating derivation from cyanidin and pelargonidin cores, respectively, with varying degrees of malonylation, feruloylation, p-coumaroylation, and caffeoylation [24]. The three most abundant anthocyanins were assigned as pelargonidin-3-O-diglucoside-5-O-glucoside (peaks 14 and 16) and pelargonidin-3-O-diglucoside (peak 17), acylated with diverse aromatic or aliphatic acids.

### 3.2. Effects of RRA Extract on Fat-Induced Aging Phenotypes

**Figure 2:**
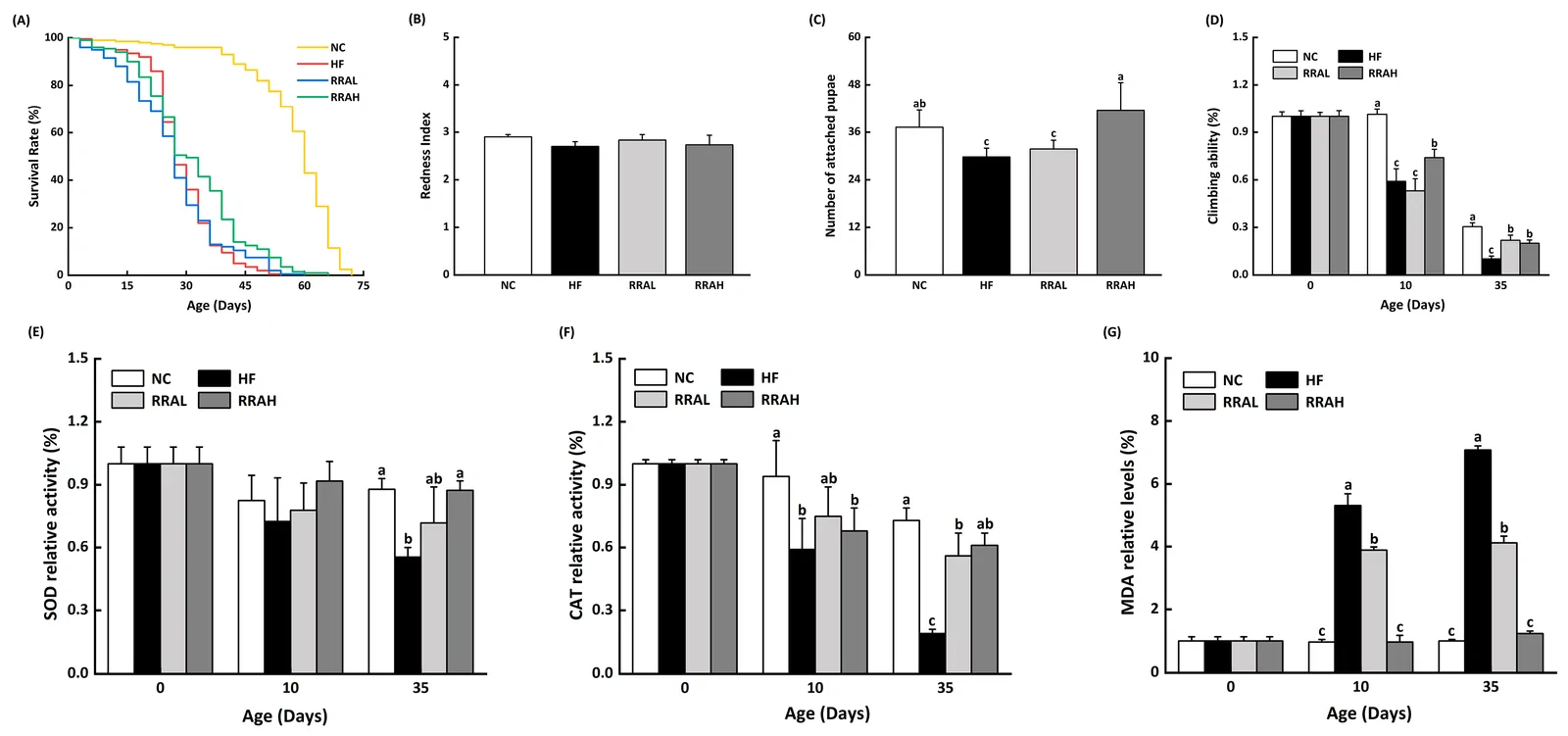
Effects of radish red anthocyanin (RRA) extract on high fat-induced aging phenotypes in *Drosophila melanogaster*. (**A**) Lifespan. (**B**) Stomach redness index. (**C**) Reproductive capacity. (**D**) Climbing ability. (**E**) Superoxide dismutase (SOD) capacity. (**F**) Catalase (CAT) capacity. (**G**) Malondialdehyde (MDA) level. NC, standard non-fat diet; HF, high-fat diet; RRAL, high-fat diet supplemented with 5 mg/mL RRA extract; RRAH, high-fat diet with supplemented 10 mg/mL RRA extract. Different letters indicate significant differences (*p* < 0.05).

To evaluate the effects of RRA extract on high-fat diet-associated physiological decline, lifespan and aging-related phenotypes were assessed in *Drosophila melanogaster*. Compared with NC group, high-fat feeding markedly shortened median survival time, maximum lifespan, and mean lifespan of fruit flies (Table 1 & Figure 2A), indicating supplementation of 10% lard adversely affected survival. RRA extract partially attenuated these effects, with greater improvements observed at the high dose. Compared with HF group, RRAH group showed increases of 11% in median survival time and 24% in mean maximum lifespan (Table 1).

The survival benefits were accompanied by improvements in physiological performance. No significant difference was observed in the abdominal redness index among groups (Figure 2B), indicating the observed benefits were not accompanied by a detectable reduction in food intake in this assay [2]. High-fat feeding also impaired reproductive performance and climbing ability, whereas RRA supplementation partially attenuated these impairments (Figure 2C,D). The improvements in survival and functional performance supported a protective effect of RRA extract against high-fat diet-associated physiological decline.

High-fat feeding was accompanied by lower antioxidant enzyme activities and greater lipid peroxidation, consistent with disturbed redox homeostasis [25,26]. In RRA treatment groups and the measurements on days 10 and 35, SOD and CAT activities increased by 7–58% and 17–222%, respectively, however MDA levels decreased by 27–83% relative to the corresponding HF values (Figure 2E–G). These changes were consistent with enhanced antioxidant defenses and reduced lipid peroxidation. Similar associations between anthocyanin supplementation and improved antioxidant status have been reported previously [11].

### 3.3. Effects of RRA Extract on Fat-Induced Stress Challenge

**Figure 3:**
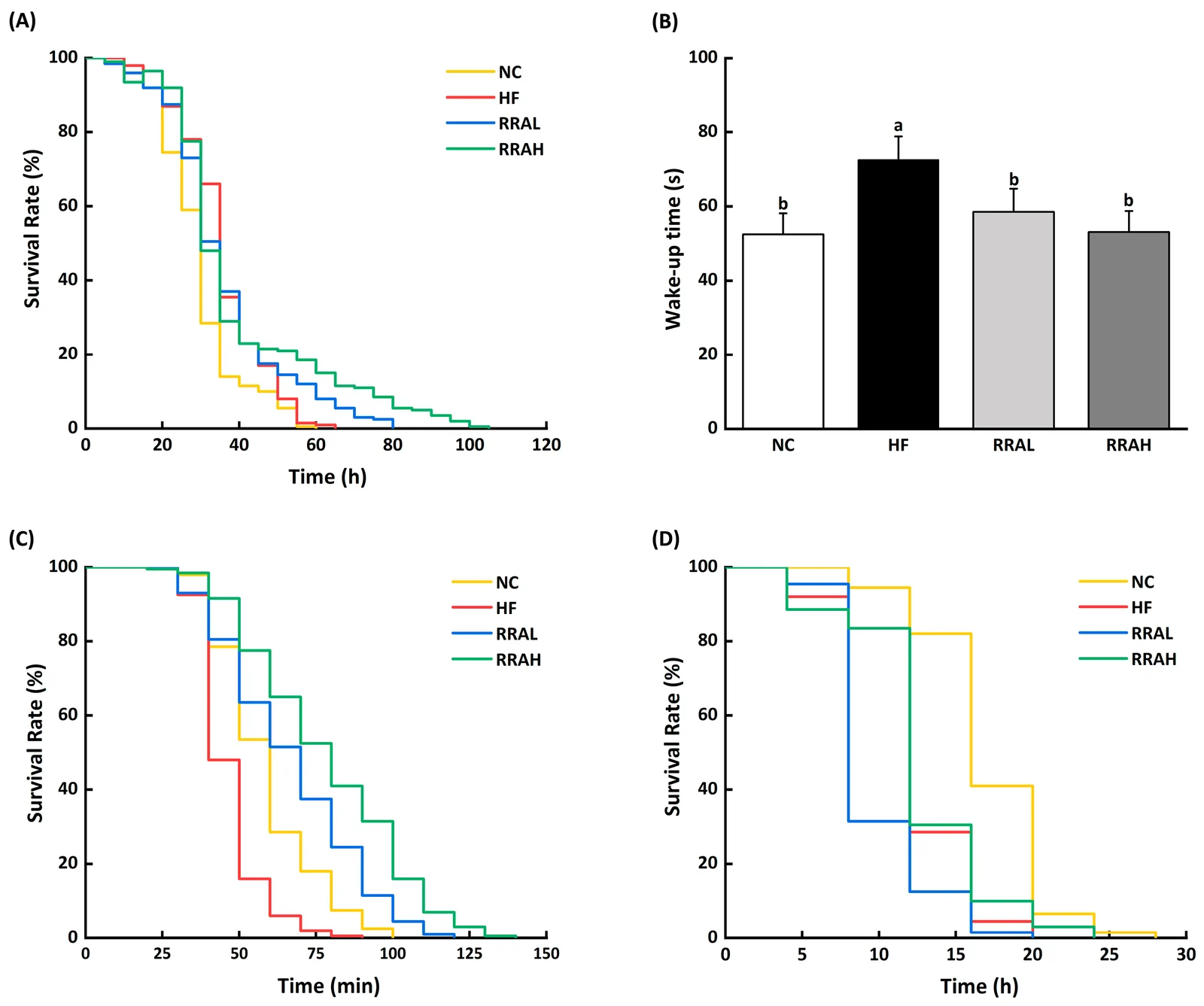
Effects of radish red anthocyanin (RRA) extract on high-fat diet-induced changes in stress resistance in *Drosophila melanogaster*. (**A**) Starvation stress resistance. (**B**) Cold shock recovery. (**C**) Heat stress resistance. (**D**) Oxidative stress resistance. NC, standard non-fat diet; HF, high-fat diet; RRAL, high-fat diet supplemented with 5 mg/mL RRA extract; RRAH, high-fat diet supplemented with 10 mg/mL RRA extract. Different letters indicate significant differences (*p* < 0.05).

Resistance to environmental challenges provides an additional measure of physiological function. High-fat feeding increased survival under starvation (Figure 3A), but impaired responses to cold shock (Figure 2B), heat shock (Figure 3C), and H_2_O_2_ exposure (Figure 3D). These responses indicated that high-fat feeding affected stress tolerance in a challenge-dependent manner. Greater starvation resistance may reflect altered energy reserves or energy expenditure rather than an overall improvement in physiological condition. RRA supplementation improved the measured survival or recovery outcomes under starvation, cold, and heat stress. These findings broadened the evidence for its protective effects beyond lifespan extension and suggested improved tolerance to selected environmental challenges.

### 3.4. Effects of RRA Extract on Fat-Induced Intestinal Damage

**Figure 4:**
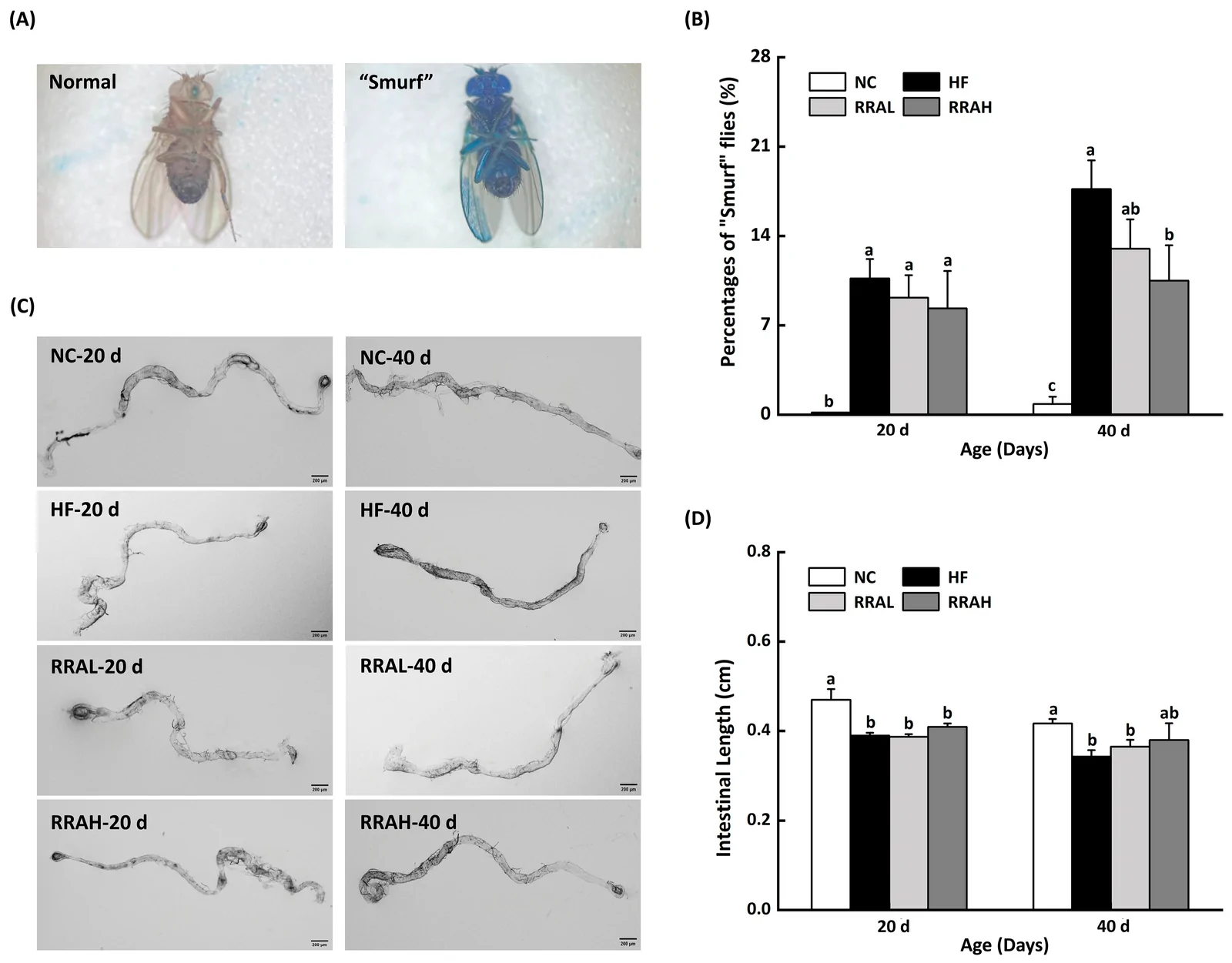
Effects of radish red anthocyanin (RRA) extract on high-fat diet-induced intestinal barrier damage in *Drosophila melanogaster*. (**A**) Representative images of normal and “Smurf” flies after Brilliant Blue FCF feeding. (**B**) Percentage of ”Smurfs”flies in different groups. (**C**) Representative images of dissected guts. (**D**) Intestinal length. NC, standard non-fat diet; HF, high-fat diet; RRAL, high-fat diet supplemented with 5 mg/mL RRA extract; RRAH, high-fat diet supplemented with 10 mg/mL RRA extract. Different letters indicate significant differences (*p* < 0.05).

“Smurf” assay and gut length measurements was used to evaluate intestinal integrity. Following Brilliant Blue FCF feeding, flies displaying blue coloration of the digestive tract were defined as “Smurf”, indicating loss of intestinal barrier integrity (Figure 4A). High-fat feeding increased the proportion of “Smurf” flies, while the age-associated increase in this proportion indicated progressive deterioration of barrier function (Figure 4B). RRA supplementation markedly decreased the percentage of “Smurf” flies only on day 40, suggesting that its detectable protective effect was most evident at the later age examined. High-fat feeding also shortened the intestine (Figure 4C,D), consistent with previous reports of intestinal damage under high-fat dietary conditions [27]. However, RRA supplementation did not significantly restore gut length (Figure 4D). Thus, its effect on intestinal leakage was not accompanied by a demonstrable recovery of this morphological measure. These results supported a selective benefit for barrier integrity rather than complete prevention of intestinal damage.

### 3.5. Effects of RRA Extract on Fat-Induced Gene Expression

**Figure 5:**
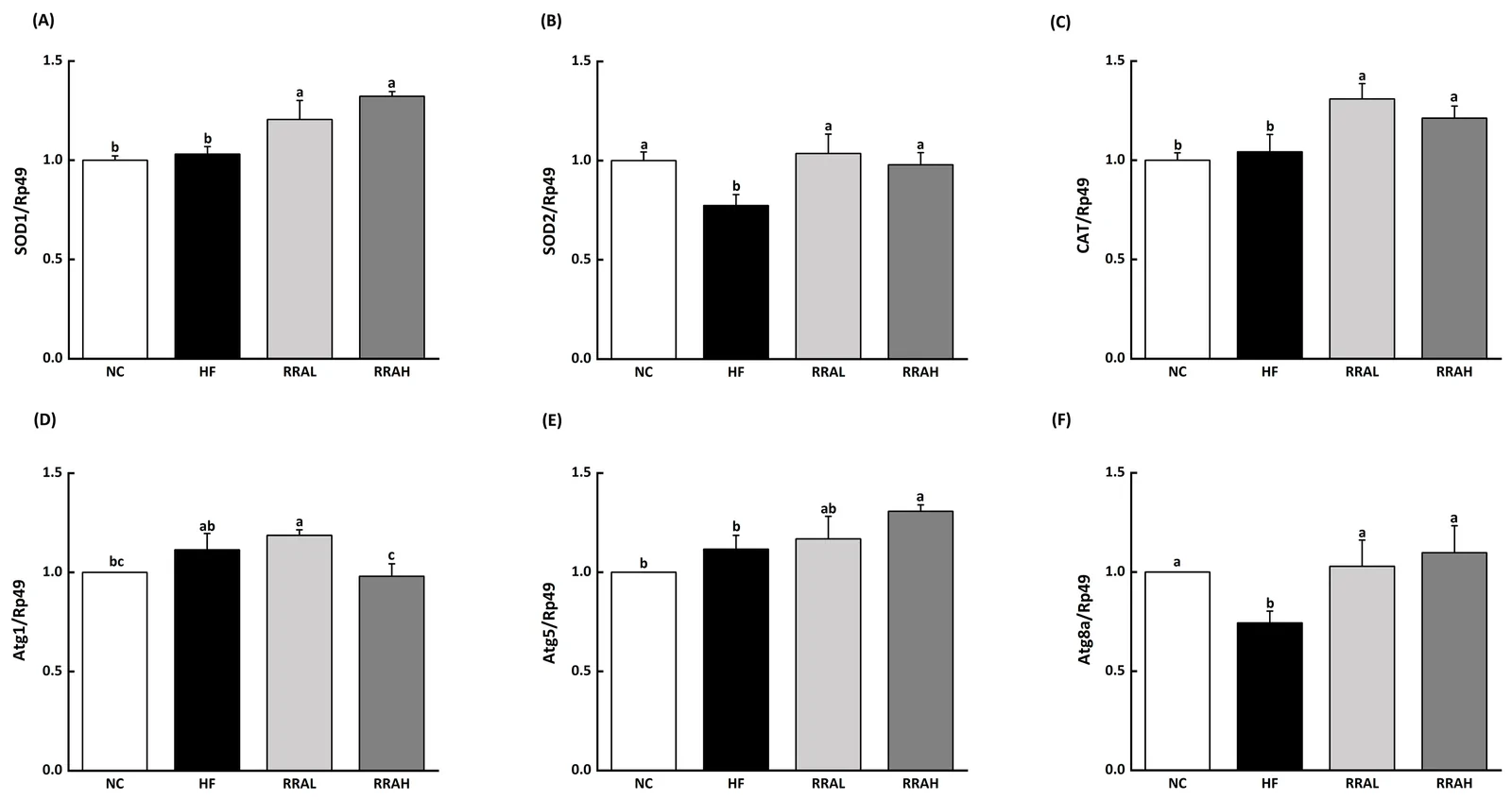
Effects of radish red anthocyanins (RRA) extract on high-fat diet-induced changes in the mRNA expression levels of antioxidant- and autophagy-related genes in *Drosophila melanogaster.* (**A**) Copper-zinc superoxide dismutase (SOD1). (**B**) Manganese superoxide dismutase (SOD2). (**C**) Catalase (CAT). (**D**) Autophagy-related gene 1 (Atg1). (**E**) Autophagy-related gene 5 (Atg5). (**F**) Autophagy-related gene 8a (Atg8a). NC, standard non-fat diet; HF, high-fat diet; RRAL, high-fat diet supplemented with 5 mg/mL RRA extract; RRAH, high-fat diet supplemented with 10 mg/mL RRA extract. Different letters indicate significant differences (*p* < 0.05).

Antioxidant- and autophagy-related gene expression was examined to explore molecular responses associated with the protective effects of RRA extract. Compared with NC group, high-fat feeding significantly decreased SOD2 expression but did not significantly alter SOD1 or CAT expression (Figure 5A–C). RRA supplementation increased SOD1 and CAT expression and restored SOD2 expression. These transcriptional responses were broadly consistent with the higher SOD and CAT activities observed following RRA supplementation (Figure 5E,F).

High-fat feeding also affected autophagy-related transcription. Expression of Atg1 and Atg5 was not significantly decreased, whereas Atg8a expression was markedly reduced in HF group compared to NC group (Figure 5D–F). RRA supplementation restored Atg8a expression to a level comparable to that in NC group and increased Atg5 expression. These findings indicated that RRA extract influenced the transcriptional responses of selected autophagy-related genes under high-fat feeding. Autophagy contributes to cellular maintenance by removing damaged proteins and organelles, and its dys-regulation is implicated in aging-related functional decline [28]. The combined biochemical and transcriptional results suggested RRA-associated improvements in physiological performance were accompanied by changes in antioxidant defenses and cellular maintenance-related responses [29]. Related protective effects have been reported for hawthorn extract [30], black rice anthocyanins [11], purple sweet potato anthocyanins [2], and blackcurrant extract [12].

### 3.6. Effects of RRA Extract on Fat-Induced Changes of Gut Microbiota

#### 3.6.1. Alteration in Gut Bacterial Diversity

**Figure 6:**
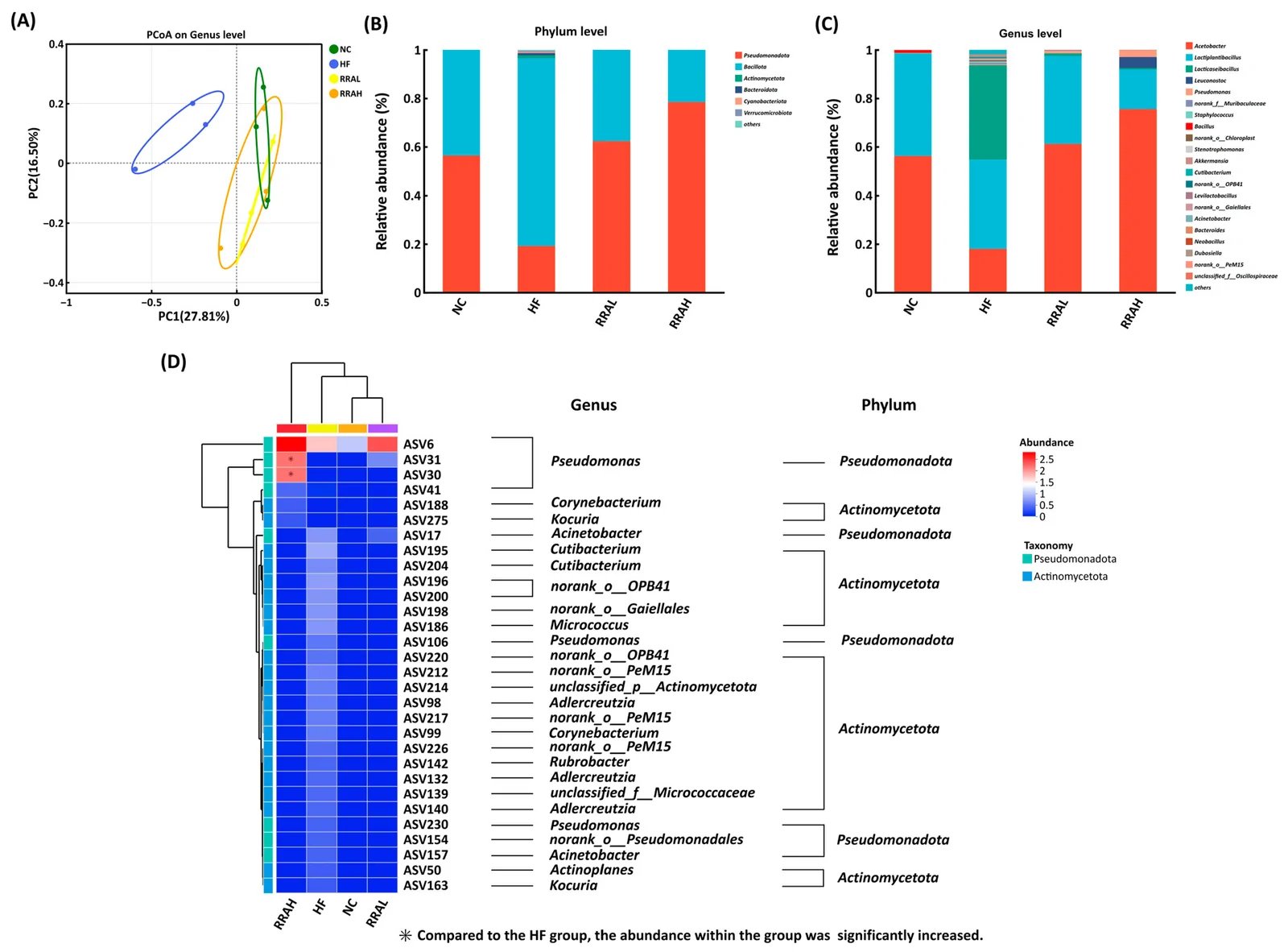
Effects of radish red anthocyanin (RRA) extract on high-fat diet-induced changes of gut microbiota composition in *Drosophila melanogaster*. (**A**) Principal coordinate analysis (PCoA) of gut microbial communities. (**B**) Microbial composition at the phylum level. (**C**) Microbial composition at the genus level. (**D**) Heatmap showing the relative abundance of 30 ASVs in the fly gut microbiota. Asterisk indicates significant differences in abundance (*p* < 0.05), and the color gradient from blue to red represents low to high relative abundance. NC, standard non-fat diet; HF, high-fat diet; RRAL, high-fat diet supplemented with 5 mg/mL RRA extract; RRAH, high-fat diet supplemented with 10 mg/mL RRA extract.

After quality filtering, 498,048 high-quality sequencing reads were retained, and 277 amplicon sequence variants (ASVs) were identified. Rarefaction curves reached a plateau, suggesting sequencing depth was adequate to identify predominant bacterial diversity across the samples (Supplementary Figure S1). HF group showed higher Sobs, ACE, and Chao richness estimates than the NC and RRA-supplemented groups (Supplementary Table S6). Increased richness alone does not establish improved microbial homeostasis. Its biological significance depends on the taxa involved and their interactions with the host [22]. Venn diagrams indicated six ASVs were shared among all four groups (Supplementary Figure S2). PCoA revealed visual separation between the NC and HF groups, while the RRA-supplemented groups occupied positions closer to NC than HF in the displayed ordination (Figure 6A). This pattern suggested treatment-associated differences in microbial community structure. However, the microbiota analysis included only three biological replicates per group, limiting statistical power and making the observed patterns potentially sensitive to individual samples. Adequate sequencing depth does not compensate for limited biological replication. These results therefore must regarded as exploratory and need further validation using larger independent cohorts.

#### 3.6.2. Alterations in Taxonomic Composition and Distinct Bacterial Taxa

*Pseudomonadota* and *Bacillota* were the major bacterial phyla among all samples (Figure 6B). High-fat feeding lowered the relative amount of *Pseudomonadota* and boosted that of *Bacillota*, whereas RRA supplementation partially reversed these compositional changes. These results indicated a dietary treatment-associated shift in bacterial composition. Under such conditions, redox-sensitive members within *Pseudomonadota* might be suppressed, whereas *Bacillota* with greater stress adaptability might gain a competitive advantage [22,31].

At the genus level, *Acetobacter* and *Lactiplantibacillus* predominated in the NC group. High-fat feeding was related to a reduced relative amount of *Acetobacter* and an increased relative amount of *Lacticaseibacillus* (Figure 6C). RRA supplementation increased the relative representation of *Acetobacter*, shifting this aspect of community composition toward NC pattern. Since *Acetobacter* is a major genera in the healthy intestinal microbiota of flies and involved in growth [32], nutrient utilization [33], longevity [34], and detoxification [35,36], its reduction in HF group might indicate a disruption of the normal microbial structure. By contrast, the enrichment of *Lacticaseibacillus* under high-fat feeding might represent an adaptive expansion of stress-tolerant taxa in response to fat-induced intestinal stress, rather than a simple beneficial shift [22,37].

LEfSe analysis identified five discriminatory taxonomic features (Supplementary Figure S3). *Actinomycetota* was enriched in HF group, whereas *Pseudomonadales*, *Pseudomonadaceae*, *Pseudomonas*, and unclassified *Pseudomonastaxon* were enriched in the RRAH group. An ASV-level heatmap demonstrated the amount patterns of selected bacterial features (Figure 6D). ASV30 and ASV31, both assigned to *Pseudomonas*, showed increased relative amount in RRAH group compared to HF group (*p* < 0.05, Figure 6D). This finding identified specific bacterial features associated with high-dose RRA supplementation.

### 3.7. Effects of RRA Extract on Gut Metabolites in Drosophila Melanogaster

#### 3.7.1. Untargeted Metabolomics Analysis

**Figure 7:**
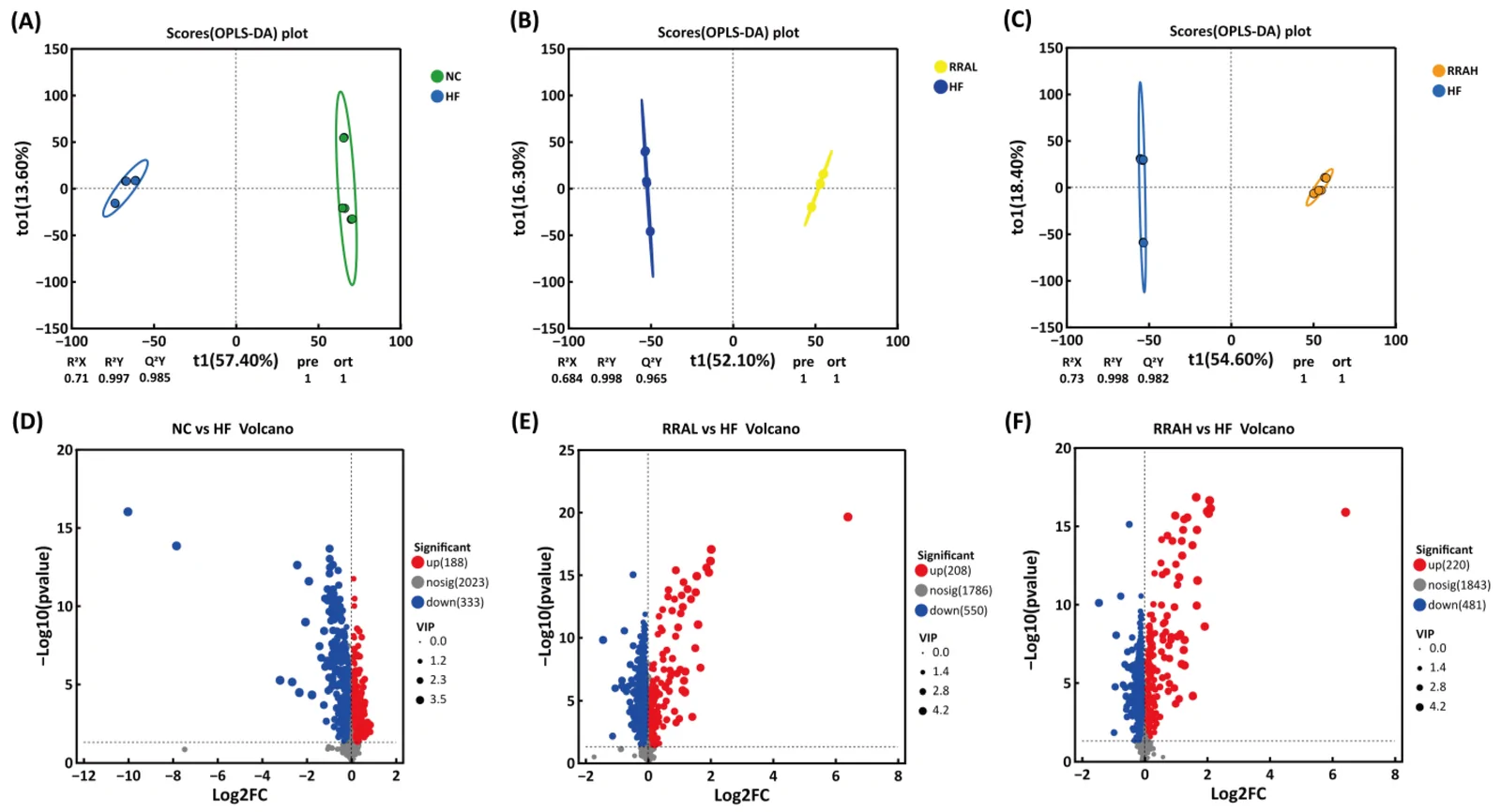
Untargeted metabolomics analysis of gut metabolites in *Drosophila melanogaster*. Orthogonal partial least squares-discriminant analysis (OPLS-DA) was performed to compare metabolite profiles among groups: (**A**) NC vs HF, (**B**) RRAL vs HF, and (**C**) RRAH vs HF. Volcano plots were generated to visualize differential metabolites based on variable importance in projection (VIP) values and statistical significance: (**D**) NC vs HF, (**E**) RRAL vs HF, and (**F**) RRAH vs HF. NC, standard non-fat diet; HF, high-fat diet; RRAL, high-fat diet supplemented with 5 mg/mL radish red anthocyanins extract; RRAH, high-fat diet supplemented with 10 mg/mL radish red anthocyanins extract.

Intestinal metabolic changes fed with high-fat diet and RRA supplementation was analyzed using Untargeted metabolomics. A total of 1500 metabolites were reported from analyzed ionization modes. PCA showed separation among all groups, indicating differences in intestinal metabolite profiles (Supplementary Figure S4). OPLS-DA further discriminated HF group from NC, RRAL, and RRAH groups (Figure 7A–C). The high R^2^X, R^2^Y, and Q^2^, together with permutation test results (Supplementary Figure S5), indicated the models had good explanatory and predictive ability without obvious overfitting. Based on the OPLS-DA models, 521 metabolites differed in the NC-versus-HF comparison, comprising 188 with higher and 333 with lower relative abundances in NC (Figure 7D). Relative to HF, RRAL group showed 208 higher-abundance and 550 lower-abundance metabolites, while RRAH group showed 220 higher-abundance and 481 lower-abundance metabolites (Figure 7E,F). These findings indicated substantial treatment-associated differences in intestinal chemistry.

#### 3.7.2. Pathway Enrichment and Clustering of Differential Metabolites

**Figure 8:**
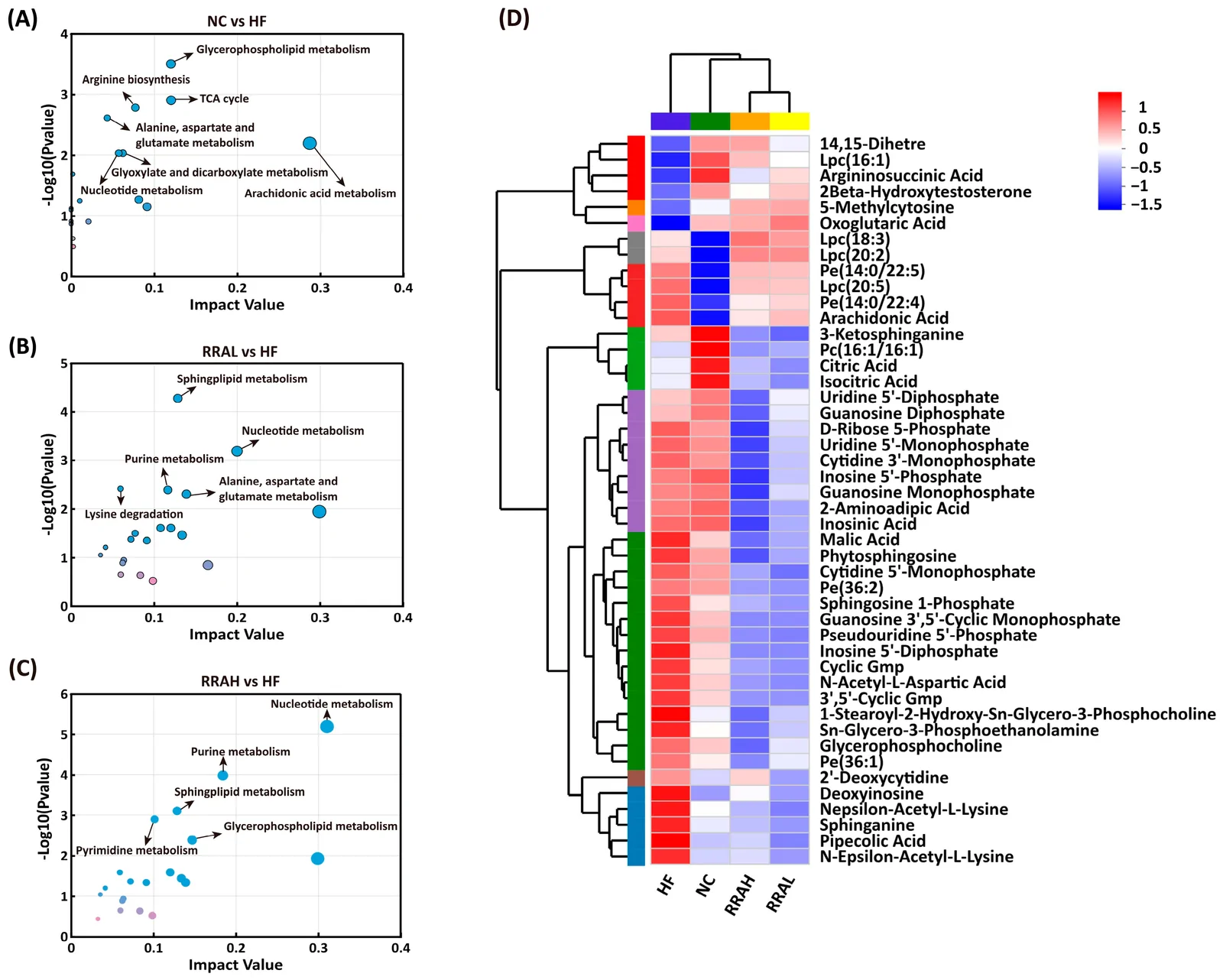
Kyoto Encyclopedia of Genes and Genomes (KEGG) pathway impact analysis of differential metabolites is shown for (**A**) NC vs HF, (**B**) RRAL vs. HF, and (**C**) RRAH vs. HF. Each bubble represents a metabolic pathway; bubble size indicates the pathway impact value, and the y-axis represents pathway enrichment significance -log_10_(p). Pathways with adjusted *p* values < 0.05 are marked with black arrows. (**D**) Hierarchical clustering analysis of differential metabolites. Each column represents an individual sample, each row represents a differential metabolite, and the color gradient indicates the relative abundance of metabolites across samples. NC, standard non-fat diet; HF, high-fat diet; RRAL, high-fat diet supplemented with 5 mg/mL radish red anthocyanins extract; RRAH, high-fat diet supplemented with 10 mg/mL radish red anthocyanins extract.

In NC-versus-HF comparison, KEGG enrichment analysis identified pathways involved in lipid, energy, amino acid, and nucleotide metabolism (Figure 8A, adjusted *p* value < 0.01). Figure 8D showed higher relative abundances of several glycerophospholipid-, sphingolipid-, and nucleotide-related metabolites in HF group than in NC group based on hierarchical clustering. Glycerophospholipids are major membrane constituents [38], while sphingolipids participate in membrane organization and cellular signaling [39]. Differences in these metabolites were therefore consistent with altered intestinal lipid homeostasis under high-fat feeding.

The RRAL-versus-HF and RRAH-versus-HF comparisons identified enriched pathways involving sphingolipid, glycerophospholipid, nucleotide, and amino acid metabolism (Figure 8B,C, adjusted *p* value < 0.01). Clustering showed RRA supplementation generated profiles distinct from those of HF group, rather than uniformly returning all metabolites to NC levels (Figure 8D) [40]. Thus, RRA supplementation was associated with a modification of the intestinal metabolic response to high-fat feeding.

#### 3.7.3. Integrated Correlation Analysis

Associations among selected bacterial ASVs, differential metabolites, and stress-resistance indicators were identified by spearman correlation analysis (Supplementary Figure S6). Interpretation focused on ASV30 and ASV31 because their relative abundances were increased in RRAH group compared to HF group (Figure 6D). ASV30 and ASV31 were markedly negatively correlated with selected nucleotide-related metabolites, including uridine 5′-monophosphate and guanosine monophosphate (Supplementary Figure S6). Both ASVs showed positive correlations with indicators of starvation and heat-stress resistance (Supplementary Figure S6, *p* < 0.01). These associations indicated RRAH-associated bacterial changes occurred alongside differences in intestinal metabolites and stress responses. However, correlations across treatment groups might reflect shared responses to dietary treatment rather than direct microbial regulation of host metabolism. They therefore did not establish that these *Pseudomonas*-related ASVs caused the metabolic changes or mediated the protective effects of RRA. Furthermore, microbiota and metabolomic profiles were assessed only on day 20, preventing determination of their temporal relationships with physiological changes. Longitudinal sampling, targeted metabolite quantification, and microbial depletion or reconstitution experiments are needed to clarify these relationships and assess causality.

## 4. Conclusions

RRA extract supplementation attenuated several high-fat diet-associated aging phenotypes in *Drosophila melanogaster*, including shortened lifespan and impaired reproductive and locomotor performance. These benefits were accompanied by increased SOD and CAT activities, lower MDA levels, and improved responses to starvation, cold, and heat stress. RRA extract also reduced intestinal leakage at day 40, although gut length was not significantly restored. Changes in antioxidant- and autophagy-related gene expression indicated associated transcriptional responses but did not establish increased autophagic flux. Gut microbiota and metabolomic analyses revealed treatment-associated differences in bacterial composition and intestinal metabolites.

Our observations were helpful in further exploring RRA extract as a food-derived bioactive preparation for preserving physiological function under high-fat feeding. However, the absence of an RRA-supplemented standard-diet group prevents determination of whether similar benefits occur under standard feeding or depend on dietary fat exposure. A factorial design incorporating RRA supplementation under both dietary conditions is needed to address this question. Lifespan and most physiological assessments were also conducted in male flies of a single strain, limiting generalization to females and other genetic backgrounds. Furthermore, the extract-based intervention does not resolve the contributions of individual anthocyanins, and no conclusions about relative efficacy can be drawn without comparisons with other anthocyanin preparations. Differences in physiology and dietary compound metabolism between flies and humans further limit direct extrapolation. Mechanistic validation and evaluation of efficacy, safety, bioavailability, and achievable dietary exposure in mammalian models are therefore needed before considering applications in human nutrition.

## Figures and Tables

**Table 1:** Effects of radish red anthocyanin (RRA) extract on high-fat diet-induced lifespan in *Drosophila melanogaster*.

Group	Median Survival Time (d)	Mean Maximum Lifespan (d)	Mean Lifespan (d)
NC	55.19 ± 5.22 ^a^	69.3 ± 2.21 ^a^	57.48 ± 5.66 ^a^
HF	26.55 ± 2.43 ^bc^	45.1 ± 5.56 ^c^	28.74 ± 2.31 ^b^
RRAL	24.23 ± 2.95 ^c^	47.1 ± 7.75 ^c^	26.91 ± 5.31 ^b^
RRAH	29.73 ± 7.64 ^b^	55.8 ± 6.66 ^b^	31.20 ± 5.45 ^b^

NC, standard non-fat diet; HF, high-fat diet; RRAL, high-fat diet supplemented with 5 mg/mL RRA extract; RRAH, high-fat diet supplemented with 10 mg/mL RRA extract. Different letters indicate significant differences (*p* < 0.05).

## Data Availability

Data will be made available on request.

## Supplementary Materials

The following supporting information can be downloaded at: https://www.mdpi.com/article/10.3390/nu18213499/s1, Figure S1: Rarefaction analysis of gut microbiota in *Drosophila melanogaster*. (A) Sobs index. (B) Shannon index. NC, standard non-fat diet; HF, high-fat diet; RRAL, high-fat diet supplemented with 5 mg/mL radish red anthocyanins extract; RRAH, high-fat diet supplemented with 10 mg/mL radish red anthocyanins extract; Figure S2. Venne diagram showing the unique and shared amplicon sequence variants levels (ASVs) of gut microbiota in *Drosophila melanogaster*. Numbers in the overlapping regions indicate shared ASVs among groups. NC, standard non-fat diet; HF, high-fat diet; RRAL, high-fat diet supplemented with 5 mg/mL radish red anthocyanins extract; RRAH, high-fat diet supplemented with 10 mg/mL radish red anthocyanins extract; Figure S3. Linear discriminant analysis (LDA) coupled with effect size (LEfSe) analysis of gut microbiota in *Drosophila melanogaster*. NC, standard non-fat diet; HF, high-fat diet; RRAL, high-fat diet supplemented with 5 mg/mL radish red anthocyanins extract; RRAH, high-fat diet supplemented with 10 mg/mL radish red anthocyanins extract. Threshold of LDA ≥ 2.0; Figure S4. Principal component analysis (PCA) for gut metabolites in *Drosophila melanogaster*. NC, standard non-fat diet; HF, high-fat diet; RRAL, high-fat diet supplemented with 5 mg/mL radish red anthocyanins extract; RRAH, high-fat diet supplemented with 10 mg/mL radish red anthocyanins extract. QC, pooled quality control sample; Figure S5. Permutation test results of orthogonal partial least squares discriminant analysis (OPLS-DA) for gut metabolites in *Drosophila melanogaster*. (A) NC vs HF, (B) RRAL vs HF, and (C) RRAH vs HF. NC, standard non-fat diet; HF, high-fat diet; RRAL, high-fat diet supplemented with 5 mg/mL radish red anthocyanins extract; RRAH, high-fat diet supplemented with 10 mg/mL radish red anthocyanins extract; Figure S6. Spearman’s correlation heatmap among 30 key ASVs, differential metabolites, and stress resilience indicators. The color gradient represents Spearman’s correlation coefficients. Asterisks indicate statistical significance: * *p* < 0.05 and ** *p* < 0.01, and *** *p* < 0.001. These metabolites were derived from the significantly enriched metabolic pathways shown in Figure 8A–C (adjusted *p* value < 0.05). NC, standard non-fat diet; HF, high-fat diet; RRAL, high-fat diet supplemented with 5 mg/mL radish red anthocyanins extract; RRAH, high-fat diet supplemented with 10 mg/mL radish red anthocyanins extract; Table S1. Gradient elution program for UPLC-MS/MS analysis of radish red anthocyanin extract. Table S2: Details of experimental assays in Drosophila melanogaster maintained on NC, HF, RRAL, and RRAH diets, respectively; Table S3. Primer sequences of quantitative real-time polymerase chain reaction analysis; Table S4. Gradient elution program for LC-MS/MS analysis of gut metabolites; Table S5. The mass spectrometry data and identification of compounds in radish red anthocyanin extract; Table S6. Alpha diversity of gut microbiota in *Drosophila melanogaster* at the amplicon sequence variants (ASVs) levels.
