# Supplementary material for: Radish Red Anthocyanins Extract Attenuated High-Fat Diet-Induced Aging in Association with Improving Antioxidant Defense and Gut Microbiota–Metabolite Changes in Drosophila Melanogaster
Source: Nutrients. 2026 Oct 24;18(21):3499. doi: 10.3390/nu18213499 (PMC13765094; doi:10.3390/nu18213499)
Supplement: Supplementary file 1 [file nutrients-18-03499-s001.zip › nutrients-4602419-supplementary.pdf]

Table S1. Gradient elution program for UPLC-MS/MS analysis of radish red anthocyanin extract.

Time (min)	Mobile phase A (%)	Mobile phase B (%)
0	90	10
7	50	50
11	10	90
15	90	10
17	90	10

Table S2. Details of experimental assays in *Drosophila melanogaster maintained* on NC, HF, RRAL, and RRAH diets, respectively.

Assay	Number of flies per group	Age of flies	Number of vial s	Number of biological replicates	Sex of flies	Time points
**Aging Phenotypes**						
Lifespan	200	Three-day old	10	20	Male	counted every 2–3 days until day 72
Gustatory test	60	Three-day old	3	20	Male	2 h
Reproductive capacity	100	8 h of eclosion	5	20	half male and half female	on day 10
Climbing ability	200	Three-day old	10	20	Male	on days 0, 10, and 35
Antioxidant activity	200	Three-day old	10	20	Male	on days 0, 10, and 35
**Stress Challenge Tests**						
Starvation stress	200	Three-day old	10	20	Male	on day 5
Cold shock recovery	200	Three-day old	10	20	Male	on day 5
Heat stress	200	Three-day old	10	20	Male	on day 25
H_2O_2 challenge	200	Three-day old	10	20	Male	on day 5
**Intestinal Damage**						
"Smurf"	200	Three-day old	10	20	Male	on days 20 and 40
Intestinal length	200	Three-day old	10	20	Male	on days 20 and 40
**Gene expression**						
Real-time PCR	60	Three-day old	3	20	Male	on day 35
Gut Microbiota Analysis	180	Three-day old	3	60	Male	on day 20
Gut Metabolites Analysis	870	Three-day old	6	145	Male	on day 20

NC, standard non-fat diet; HF, high-fat diet; RRAL, high-fat diet supplemented with 5 mg/mL RRA extract; RRAH, high-fat diet supplemented with 10 mg/mL RRA extract.

Table S3. Primer sequences of quantitative real-time polymerase chain reaction analysis.

Genes	Primer sequences (5'-3')
rp49	Forward: GCACCAAGCACTTCATCC<br>Reverse: CGATCTCGCCGCAGTAAA
SOD1	Forward: CTGTCTGCGTAATTAACGGCGATG<br>Reverse: ATGCTCCTTGCCATACGGATTGAA
SOD2	Forward: CAAACTGCAAGCCTGGCGGTG<br>Reverse: GCTCTTCGACTTGGCCTCCTCC
CAT	Forward: CGATGCGGCTTCCAATCAGTTG<br>Reverse: ACGACACGCTCTGGAATCCG
Atg1	Forward: CGCCTTTGCCGTCGTCTACAA<br>Reverse: TGACCAGGCTGACGCAATCCT
Atg5	Forward: CACCCAGAAACTGATTTCACCGATC<br>Reverse: ATCCTTGTAGTCCACCGATAAATGC
Atg8a	Forward: CAGATGATACAGGTCATCGTCGAG<br>Reverse: GGTAGCCGATGTTGGTGGAAT

rp49, ribosomal protein 49; SOD1, copper–zinc superoxide dismutase; SOD2, manganese superoxide dismutase; CAT, catalase; Atg1, autophagy-related 1; Atg5, autophagy-related 5; Atg8a, autophagy-related 8a.

Table S4. Gradient elution program for LC-MS/MS analysis of gut metabolites.

Time (min)	Mobile phase A (%)	Mobile phase B (%)
0	100	0
0.2	100	0
3	25	25
9	0	100
10	0	100
10.1	100	0
12	100	0

Table S5. The mass spectrometry data and identification of compounds in radish red anthocyanin extract.

Peak number	t_R (min)	Prec ion (m/z)	Quantifier ion (m/z)	Qualifier ion (m/z)	Proposed structure	Content (ug/g)
1	2.68	611.20	286.9		Cyanidin-3-O-sophoroside	27.86
2	2.88	433.00	271.1		Pelargonidin-3-O-glucoside	564.85
3	3.78	595.14	271.1		Pelargonidin-3-O-sophoroside	29.00
4	3.83	935.25	286.9		Cyanidin-3-(6''-caffeylsophoroside)-5-glucoside	101.91
5	4.01	757.22	271.1		Pelargonidin-3-sophoroside-5-glucoside	2291.35
6	4.04	757.20	286.9		Cyanidin-3-O-5-O-(6-O-coumaroyl)-diglucoside	60.86
7	4.04	595.15	286.9		Cyanidin-3-O-(6-O-p-coumaroyl)-glucoside	36.13
8	4.06	919.25	271.1		Pelargonidin-3-(6''-caffeylsophoroside)-5-glucoside	2826.73
9	4.08	757.67	286.9		Cyanidin-3-O-rutinoside-5-O-glucoside	9.58
10	4.19	949.26	286.9		Cyanidin-3-O-(6''-ferulylsophoroside)-5-glucoside	112.05
11	4.40	903.26	271.1		Pelargonidin-3-O-[2-O-glucosyl-6-O-p-coumaroyl-glucoside]-5-O-glucoside	3677.95
12	4.40	741.20	271.1		Pelargonidin-3-O-5-O-(6-O-coumaroyl)-diglucoside	2073.55
13	4.41	741.67	271.1		Pelargonidin-3-O-rutinoside-5-O-glucoside	501.37
14	4.57	933.27	271.1		Pelargonidin-3-O-[6-O-feruloyl-2-O-glucosyl-glucoside]-5-O-glucoside	5235.38
15	4.71	535.11	286.9		Cyanidin-3-O-(6-O-malonyl-beta-D-glucoside)	15.49
16	5.11	989.26	271.1		Pelargonidin-3-O-(6''-ferulylsambubioside)-5-O-(malonyl)-glucoside	4160.08
17	5.23	519.43	271.1		Pelargonidin-3-O-(6-O-malonyl-beta-D-glucoside)	5116.14
18	5.65	433.39	271.1		Pelargonidin-3-O-galactoside	682.85
19	6.13	286.90	125	259.1	Dihydrokaempferol	27.11
20	6.15	271.20	215.1		Pelargonidin	12.32

tR: retention time; Prec ion, Precursor ion.

Table S6. Alpha diversity of gut microbiota in *Drosophila melanogaster* at the amplicon sequence variants (ASVs) levels.

	Sobs	ACE	Chao	Shannon	Simpson
NC	17.00±4.58^{b}	17.00±4.58^{b}	17.00±4.58^{b}	1.22±0.38	0.36±0.11
HF	65.33±45.72^{a}	65.33±45.72^{a}	65.33±45.72^{a}	1.66±0.13	0.31±0.09
RRAL	17.33±2.52^{b}	17.33±2.52^{b}	17.33±2.52^{b}	1.26±0.09	0.35±0.04
RRAH	22.33±9.07^{ab}	22.46±9.05^{ab}	22.33±9.07^{ab}	1.51±0.22	0.30±0.07

NC, standard non-fat diet ; HF, high-fat diet; RRAL, high-fat diet supplemented with 5 mg/mL red radish anthocyanin extract; RRAH, high-fat diet supplemented with 10 mg/mL red radish anthocyanin extract. Different letters indicate significant differences ($p < 0.05$).

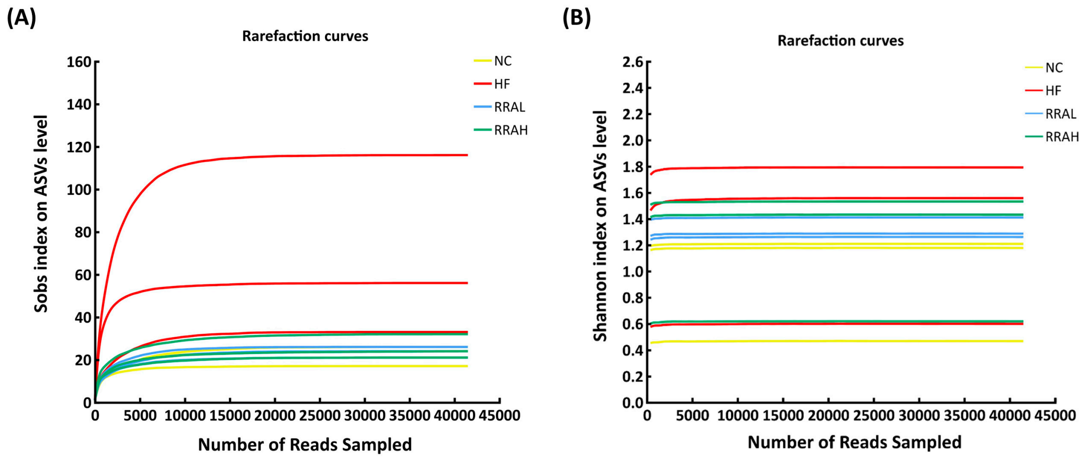

Figure S1. Rarefaction analysis of gut microbiota in *Drosophila melanogaster*. (A) Sobs index. (B) Shannon index. NC, standard non-fat diet; HF, high-fat diet; RRAL, high-fat diet supplemented with 5 mg/mL radish red anthocyanins extract; RRAH, high-fat diet supplemented with 10 mg/mL radish red anthocyanins extract.

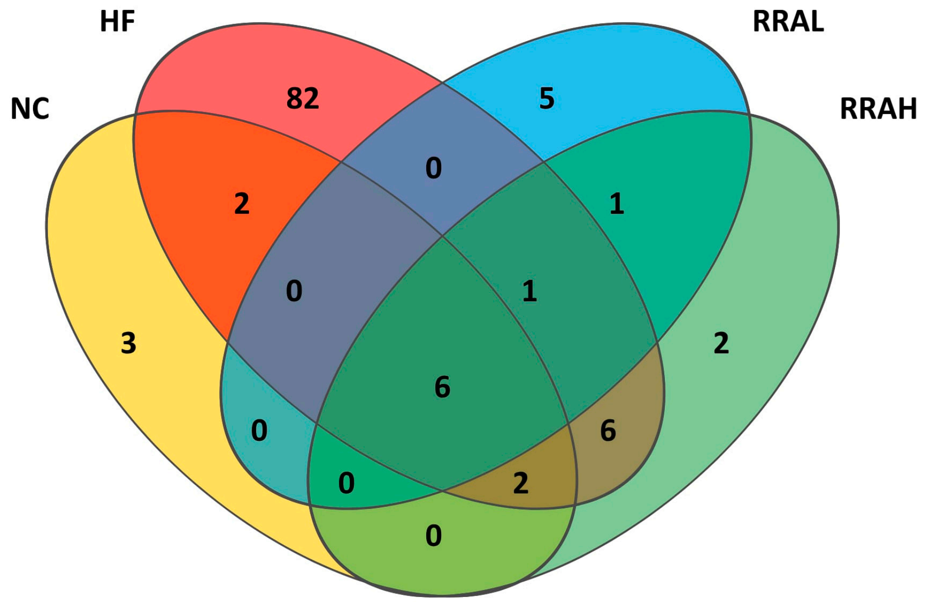

Figure S2. Venne diagram showing the unique and shared amplicon sequence variants levels (ASVs) of gut microbiota in *Drosophila melanogaster.* Numbers in the overlapping regions indicate shared ASVs among groups. NC, standard non-fat diet; HF, high-fat diet; RRAL, high-fat diet supplemented with 5 mg/mL radish red anthocyanins extract; RRAH, high-fat diet supplemented with 10 mg/mL radish red anthocyanins extract.

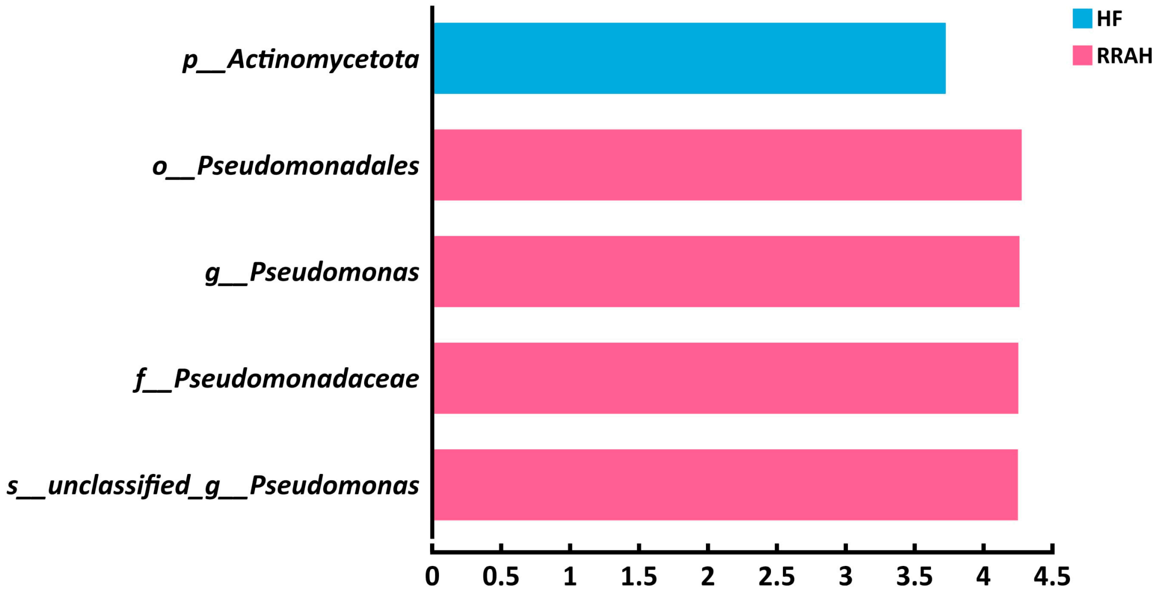

Figure S3. Linear discriminant analysis (LDA) coupled with effect size (LEfSe) analysis of gut microbiota in *Drosophila melanogaster.* NC, standard non-fat diet; HF, high-fat diet; RRAL, high-fat diet supplemented with 5 mg/mL radish red anthocyanins extract; RRAH, high-fat diet supplemented with 10 mg/mL radish red anthocyanins extract. Threshold of LDA ≥ 2.0.

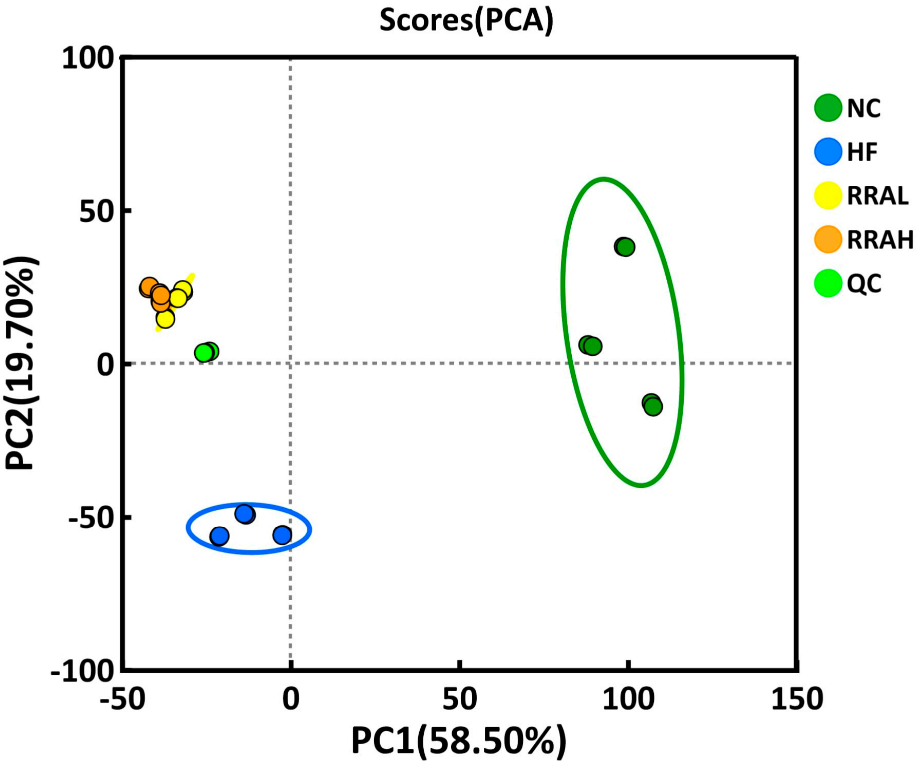

Figure S4. Principal component analysis (PCA) for gut metabolites in *Drosophila melanogaster*. NC, standard non-fat diet; HF, high-fat diet; RRAL, high-fat diet supplemented with 5 mg/mL radish red anthocyanins extract; RRAH, high-fat diet supplemented with 10 mg/mL radish red anthocyanins extract. QC, pooled quality control sample.

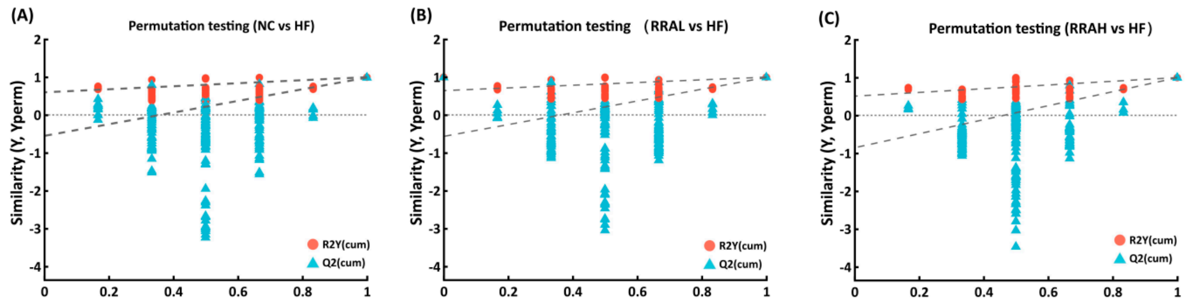

Figure S5. Permutation test results of orthogonal partial least squares discriminant analysis (OPLS-DA) for gut metabolites in *Drosophila melanogaster.* (A) NC vs HF, (B) RRAL vs HF, and (C) RRAH vs HF. NC, standard non-fat diet; HF, high-fat diet; RRAL, high-fat diet supplemented with 5 mg/mL radish red anthocyanins extract; RRAH, high-fat diet supplemented with 10 mg/mL radish red anthocyanins extract.

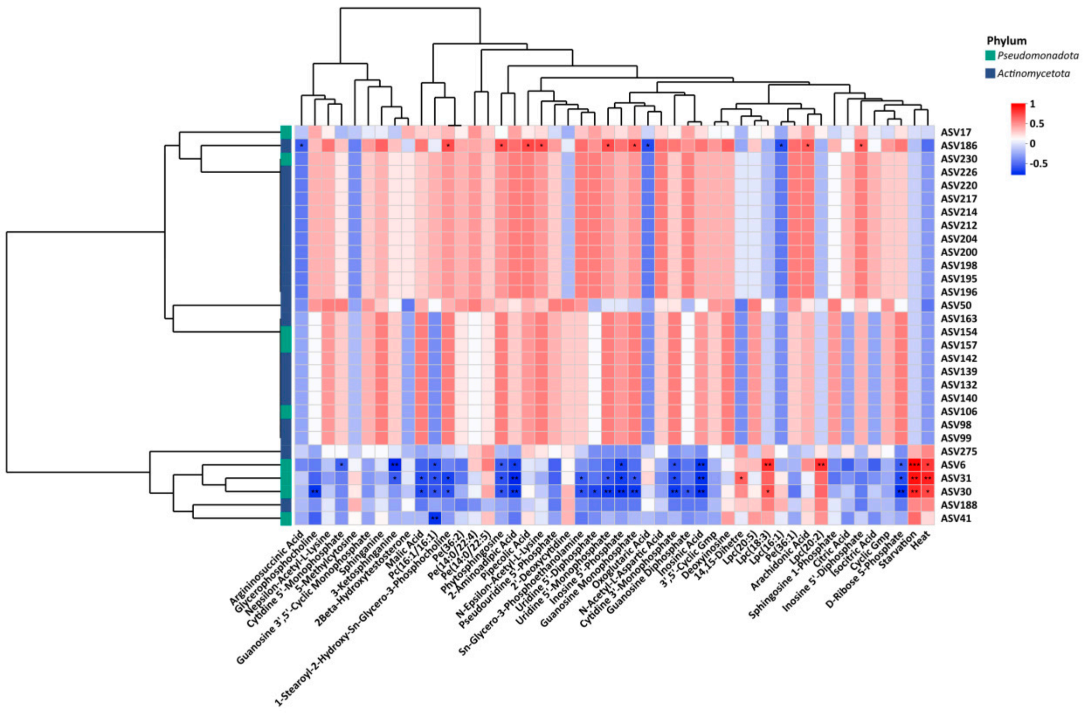

Figure S6. Spearman's correlation heatmap among 30 key ASVs, differential metabolites, and stress resilience indicators. The color gradient represents Spearman's correlation coefficients. Asterisks indicate statistical significance: **p* < 0.05 and ***p* < 0.01, and ****p* < 0.001. These metabolites were derived from the significantly enriched metabolic pathways shown in Figure 8A–C (adjusted *p* value < 0.05). NC, standard non-fat diet; HF, high-fat diet; RRAL, high-fat diet supplemented with 5 mg/mL radish red anthocyanins extract; RRAH, high-fat diet supplemented with 10 mg/mL radish red anthocyanins extract.
